# Did the post war repatriation of Lebanese physicians drive recent Lebanese medical graduates to emigrate? An observational study

**DOI:** 10.1186/1472-6963-8-195

**Published:** 2008-09-24

**Authors:** Elie A Akl, Khalil El-Asmar, Nancy Maroun, Salim M Adib, Beatrice Khater-Menassa

**Affiliations:** 1 Department of Medicine, University at Buffalo, Buffalo, USA; 2 Department of Social and Preventive Medicine, University at Buffalo, Buffalo, USA; 3 Department of Epidemiology and Population Health, American University of Beirut, Beirut, Lebanon; 4 Department of Sociology, University at Buffalo, Buffalo, USA; 5 Department of Family Medicine, Saint Joseph University, Beirut, Lebanon; 6 Department of Family Medicine, American University of Beirut, Beirut, Lebanon

## Abstract

**Background:**

A significant number of Lebanese medical graduates have emigrated from Lebanon. The objective of this study was to evaluate the hypothesis that the repatriation of Lebanese physicians educated abroad has contributed to the international emigration of recent Lebanese medical graduates.

**Methods:**

We analyzed the demographic and educational characteristics and the year of registration of physicians registered with the two physician associations in Lebanon as of 2007. We then analyzed the number of new and total registrants and the physician density for the years 1977–2006. Finally we calculated the percentage of Lebanese graduates of the years 1977–2006 registered as of 2007.

**Results:**

As of 2007, 10,918 physicians were registered in Lebanon. Most were male (80.4%) and graduated from either Lebanese (36.4%) or Eastern European (30.6%) medical schools. The top three regions of specialty training were Western Europe (31.8%), Eastern Europe (28.4%) and Lebanon (25.7%). About half the physicians registered with the Lebanese Order of Physicians as of 2007 joined during the 1990s decade; only 26.2% of these graduated from Lebanese medical schools during that decade. The number of new registrants increased dramatically in the early 1990s and started decreasing in the early 2000s. About 60% of Lebanese medical graduates of the years 1977–2006 were registered in Lebanon as of 2007. Categorizing Lebanese medical graduates by their year of graduation, the percentage registered in Lebanon as of 2007 showed a "dip" for those who graduated in the early 1990s.

**Conclusion:**

The high number of physicians educated abroad returning to Lebanon after the end of the civil war may have driven recent Lebanese medical graduates to emigrate.

## Background

The Lebanese physician workforce offers an illustrative example of the shifting dynamics of international migration of physicians. Lebanon has a physician emigration factor of 19.3%, the 7^th ^highest in the world and the highest in the Middle East and North Africa [1]. Since 1978, the number of Lebanese medical graduates practicing in the United States (US) has been consistently rising [2]. Approximately 40% of those who graduated from Lebanese medical schools over the last quarter century were active physicians in the US in 2004 [2].

A qualitative study of the factors underlying the intentions of Lebanese medical students to seek further specialty training abroad identified both push factors in Lebanon and pull factors abroad [3]. Participating medical students believed that training abroad provides them with a competitive advantage in an oversaturated Lebanese physician job market. They also thought that Lebanese citizens who had chosen medical studies and/or training abroad and then returned to Lebanon after the Lebanese civil war (1975–1990) contributed to an oversaturated physician market in Lebanon.

Daher et al. reported that in the 1990s the Lebanese physician workforce grew rapidly by 500 to 700 members per year, a rate exceeding the population growth and thereby increasing physician density [4]. Kassak et al. related this growth to three factors: the repatriation of immigrant physicians after the end of the civil war in Lebanon in 1990, the establishment of new medical schools in Lebanon, and the larger and easier enrolment of Lebanese students in the Eastern European medical schools after the breakdown of the Soviet Union [5].

The objective of our study was to assess the hypothesis that the repatriation of Lebanese physicians educated abroad has contributed to the international emigration of recent Lebanese medical graduates.

## Methods

### Evaluating the study hypothesis

Given the scarcity of workforce data in Lebanon, we explored the study hypothesis using available administrative data. We sought to understand the temporal relationship between the number of Lebanese medical graduates and physicians educated abroad practicing in Lebanon. We specifically evaluated the regions of medical school and of specialty training of the members of the Lebanese physician workforce as of 2007, the year of registration of those members, the trends of size of the workforce over the last three decades, and the membership of Lebanese medical graduates in that workforce.

### Study design

We conducted an observational study using current and historical data on physicians registered with the two Lebanese medical associations: the Lebanese Order of Physicians and the Order of Physicians in North Lebanon. The existence of two separate Orders relates to historical and political reasons going back to the creation of modern Lebanon in the 1920s. The current data consisted of the year of registration, as well as demographic and educational characteristics of registered physicians as of 2007. The historical data included the annual numbers of new and total registrants over the previous three decades (1977–2006). There was no need for ethical approval given the nature of the study.

### Data sources

The primary data sources were the databases of the two orders of physicians in Lebanon, which should include data on all practicing physicians. Indeed, to be able to practice in Lebanon, physicians have to be registered with one of these two Orders; there are mechanisms in place to avoid dual registration and to remove dead or retired physician from the databases. Physicians who are not Lebanese citizens cannot register and therefore cannot practice in Lebanon. For each registered physician, the databases report demographic characteristics (age, date of birth), educational characteristics (year of medical school graduation, country of medical school, year of completion of specialty training, country of specialty training, and type of specialty training), and contact information. Both Orders collect this information at the time of registration. The database of the Lebanese Order of Physicians contained the year of registration but unfortunately that of the Order of Physicians in North Lebanon did not.

Each Order provided the complete files of its databases in early 2007. They both also provided the annual number of new registrants and the annual total number of registrants for the time period 1977 to 2006. We categorized the variables "country of medical school" and "country of specialty training" into world regions using the World Health Organization (WHO) world regions classification while keeping Lebanon as a separate category. We merged all categories with <5% of subjects into a single "other". We also merged Northern, Southern Europe and Western Europe into a single category. We categorized the variable "training specialty" into five categories: general practice (i.e. no specialty), primary care specialty (including internal medicine, family medicine, obstetrics-gynecology and pediatrics), medical specialty, surgical specialty, and other (e.g. radiology, pathology). It is important to note that 2006 witnessed a one-month-long military conflict between Lebanon and Israel that might have delayed registration processes.

### Data analysis

First, we analyzed the demographic and educational characteristics of all physicians registered with the two orders of physicians as of 2007.

Second, we conducted multivariable analyses using consecutively the region of medical school and the region of specialty training as the dependent variables and the demographic characteristics as the independent variables. We used region of medical school (Lebanon as reference category) as an additional independent variable for region of training dependent variable. We did not use year of medical school graduation, year of completion of specialty training and year of Order registration in the regression analyses because they were significantly (p < 0.001) highly correlated with age (r = -0.96, -0.94, -0.88 respectively).

Third, we analyzed the year of registration with the Lebanese Order of Physicians of members registered as of 2007. As noted above, the Order of Physicians in North Lebanon did not contain the year of registration. We calculated the number of members that registered with the Order for each decade. We also created a graph for the number of registered physicians by year of registration for the years 1977–2006. We created similar graphs by region of medical school graduation.

Fourth, we analyzed the trend of the size of the Lebanese physician workforce over the previous three decades. We graphed the number of new registrants and the total number of registrants for the years 1977–2006. We also analyzed the trend of the physician density in Lebanon over the previous three decades. Physician density is defined as the number of physicians per 100,000 population. We obtained Lebanon's population size from the "World Population Prospects: The 2006 Revision Population Database", by the Population Division of the United Nations Department of Economic and Social Affairs [6]. The database provides revised historical population data at five years interval. We restricted our analysis of density to the years for which the number is revised. We graphed the physician density for the years 1977–2006.

Fifth, we analyzed the membership of the Lebanese medical graduates of the last three decades by the Lebanese physician workforce. We thus calculated the percentage of the graduates of Lebanese medical schools of the years 1977–2006 who were registered with one of the two Orders of physicians as of 2007. We calculated the percentage by year of graduation as well as for all years of graduation combined. We obtained the number of graduates directly from the medical schools.

We considered two-sided p values and p < 0.05 as statistically significant. We used Microsoft Excel for data management and SPSS, version 13.0 (SPSS, Inc., Chicago, Illinois), for all analyses.

## Results

### 1. General characteristics of registered physicians

As of 2007, 10,918 physicians were registered: 9,617 (88.1%) with the Lebanese Order of Physicians and 1,301 (11.9%) with the Order of Physicians in North Lebanon. Using the reported population size of 4,097,400 in 2007, the physician density would be 266 per 100,000. Table 1 shows their demographic and educational characteristics. Most were male (80.4%) and the mean age was 46.6 years (Standard Deviation (SD) = 10.8). Males were older than females (47.5 years; SD = 11.1 vs. 42.71 years; SD = 8.9; p < 0.001). The majority of registered physicians (64.8%) graduated from medical school between 1980 and 1999.

**Table 1:** Demographic and educational characteristics of physicians registered with the two orders of physicians in Lebanon as of 2007; N = 10,918.

**Age **(mean; SD)	46.6	10.8
Sex*	n	%
Female	2113	19.6%
**Year of medical school graduation**†		
Before 1970	604	5.5%
1970 – 1979	1527	14.0%
1980 – 1989	3476	31.8%
1990 – 1999	3604	33.0%
After 1999	1079	9.9%
**Region of medical school**‡		
Lebanon	3979	36.4%
Eastern Europe	3345	30.6%
Western Europe	1758	16.1%
Northern Africa	710	6.5%
Other	772	7.1%
**Year of completion of specialty training§**		
Before 1970	293	3.8%
1970 – 1979	730	9.4%
1980 – 1989	1948	25.0%
1990 – 1999	3257	41.8%
After 1999	1549	19.9%
**Region of specialty training¶**		
Western Europe	2477	31.8%
Eastern Europe	2212	28.4%
Lebanon	2008	25.7%
Northern America	617	7.9%
Other	1034	5.4%
**Type of specialty training**		
General practice (no specialty training)	3119	28.6%
Primary care specialty	2268	20.8%
Medical specialty	1883	17.2%
Surgical specialty	2438	22.3%
Other	1210	11.1%

* Data missing for n = 124 (1.1%)

† Data missing for n = 628 (5.8%)

‡ Data missing for n = 354 (3.2%)

§Data missing for n = 22 (0.3%); excluding physicians reporting general practice

¶Data missing for n = 68 (n = 0.9%); excluding physicians reporting general practice

### 2. Regions of medical school and of specialty training

The top two regions where Lebanese citizens completed medical studies were Lebanon (36.4%) and Eastern Europe (30.6%) (Table 1). In the regression analysis (using Lebanon as reference category), studies in Northern Africa, Eastern Europe, and Western Europe were associated with both older age and male sex.

The top three regions of specialty training were Western Europe (31.8%), Eastern Europe (28.4%) and Lebanon (25.7%). In the regression analysis (using Lebanon as reference category), Northern American specialty training was associated with older age and male sex and inversely associated with a medical school in Northern Africa or in Eastern Europe. Eastern Europe as the region of specialty training was associated with medical studies in Northern Africa or in Eastern Europe. Western Europe as the region of specialty training was associated with age, male sex, and a medical school in Northern Africa, Eastern Europe, or Western Europe.

### 3. Year of registration

**Figure 1:**
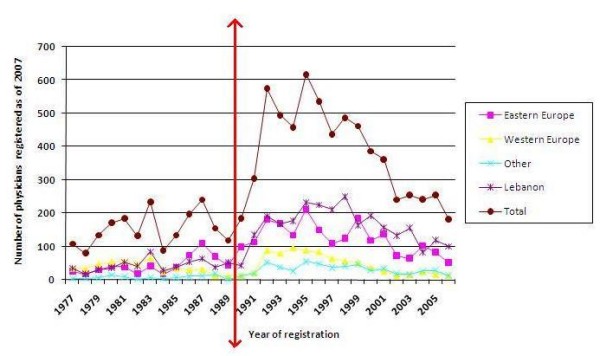
**Number of physicians registered as of 2007 with the Lebanese Order of Physician by region of medical school and by year of registration (data were not available for the Order of Physicians in North Lebanon). **The red line indicates the end of the civil war.

Figure 1 shows the trend for the year of registration with the Lebanese Order of Physicians (data were not available for the Order of Physicians in North Lebanon). Visual inspection shows a several spikes during the post-war 1990s decade. Indeed, the percentage of physicians registered by decade was 0.1% for the 1940s, 0.8% for the 1950s, 3.3% for the 1960s, 7.4% for the 1970s, 17.4% for the 1980s, 48.5% for the 1990s, and 22.4% for the 2000s. The 4467 that registered during the 1990s graduated from medical schools located mainly in the three following regions: Western Europe (13.8%), Eastern Europe (31.5%), and Lebanon (38.6% with 26.2% graduating in the 1990s).

### 4. Trends of the workforce

**Figure 2:**
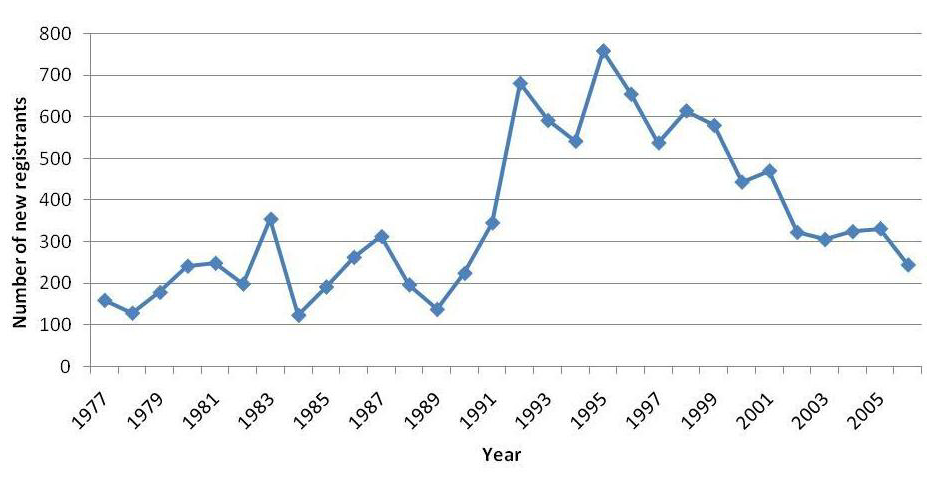
Number of physicians newly registered with the two orders of physicians in Lebanon over the 1977–2006 period.

**Figure 3:**
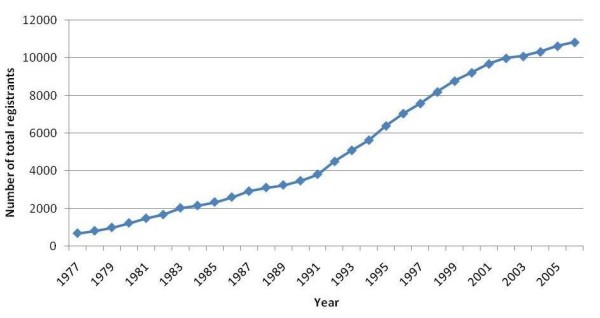
Total number of physicians registered with the two orders of physicians in Lebanon over the 1977–2006 period.

Figures 2 and 3 depict respectively the trends of the number of new registrants and the total number of registrants over the 1977–2006 period. The number of new registrants increased dramatically in the early 1990s and started decreasing in the early 2000s. In parallel, the total number of registrants showed an upward slope that became steeper in the early 1990s and then less steep in the early 2000s.

**Figure 4:**
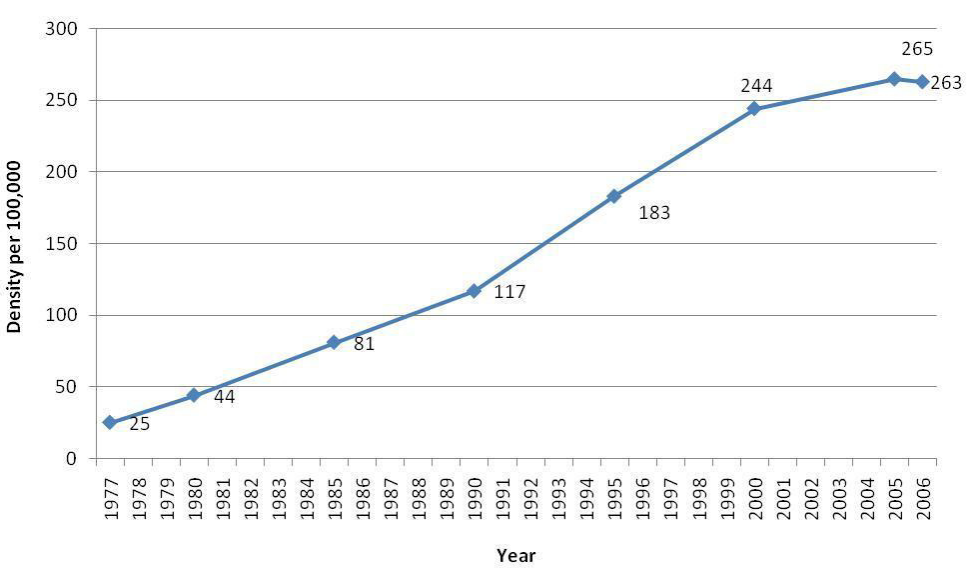
Physician density in Lebanon over the 1977–2006 period.

Figure 4 depicts the trend of the physician density in Lebanon over the 1977–2006 period. The physician density showed an upward slope since 1977 and increased by a multiple of 11 from 1977 (25 per 100,000) to 2006. Visual inspection of the curve shows that the physician density has been leveling off since the early 2000s.

### 5. Membership of Lebanese medical graduates in the workforce

**Figure 5:**
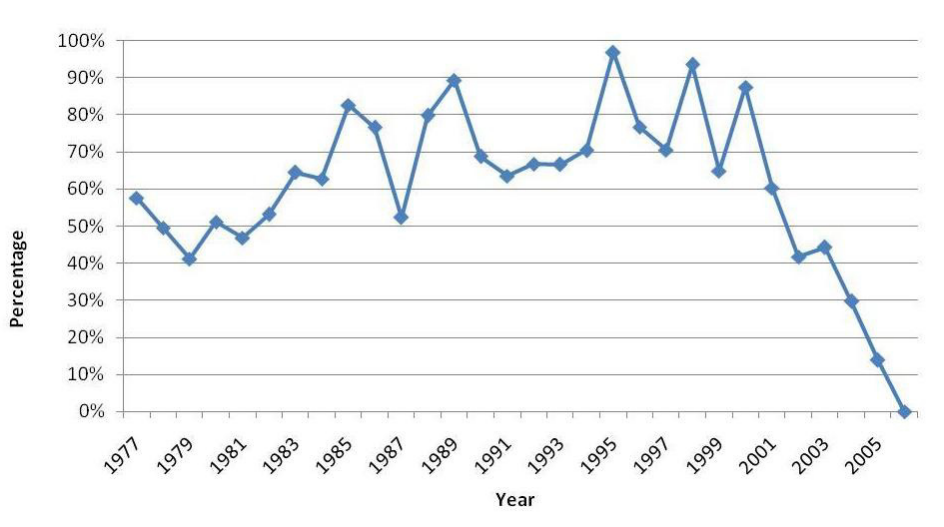
P**ercentages of Lebanese medical graduates registered with the two orders of physicians in Lebanon as of 2007, by year of graduation for the 1977–2006 period.**

Of those who graduated from Lebanese medical schools between 1977 and 2006, only 60% were registered with one of the two orders of physicians as of 2007. Visual inspection of the percentages of registration by year of graduation shows a "dip" for graduates of the early 1990s and a downward trend starting with the year 2000 (Figure 5).

## Discussion

The findings of this study support but do not confirm the hypothesis that the repatriation of Lebanese physicians educated abroad during the post-war 1990s decade has contributed to the international emigration of recent Lebanese medical graduates. In fact, the 1990s have witnessed the following concurrent trends: (1) a dramatic increase in the number of new registrants in the early 1990s; (2) about half of the physicians registered with the Lebanese Order of Physicians joined it during the 1990s decade; (3) only a quarter of those who joined the 1990s decade graduated from Lebanese medical schools during that decade; (4) a "dip" in the percentage of Lebanese graduates of the early 1990s registered with the two Orders as of 2007; (5) a "bump" in the number of Lebanese medical graduates registered as residents in the US in the early 1990s [2]. These conclusions are obviously weakened by the potential confounding by other factors such as the civil disturbances in the late 1980s.

Figure 1 suggests three sources for the growth of the number of registered physicians: graduates of Western Europe who may have returned after the end of civil war (post-war 1990s decade); graduates of Eastern Europe whose numbers appear to have increased after the 1990s dissolution of the Soviet Union and the liberalization of medical recruitment; and the increasing numbers of Lebanese medical graduates (steady increase from 99 in 1977 to 288 in 2006 with a small drop in the late 1990s; unpublished data).

There is an apparent discrepancy between the increasing number of Lebanese graduates joining the workforce in the 1990s and the "dip" in the percentage of Lebanese graduates of the early 1990s registered as of 2007. This could be explained by that most Lebanese graduates joining the workforce in 1990s likely graduated in previous decades and had been in migrant status before their return. Their increasing influx from other countries consequently pushed Lebanese graduates of the early 1990s to get out of Lebanon.

This study has a number of strengths. First, it covers a time span of three decades allowing the depiction of long term trends for the number of new and total registered physicians and for physician density. Second, it analyzes the characteristics of all physicians registered in Lebanon. Third, the two Orders collect the data directly from registrants and require official documentation (e.g., diplomas, certificates) to verify the educational credentials, which strengthens the validity of the data. Finally, we were not able to identify published literature on a similar phenomenon of repatriation of physicians driving the emigration of recent graduates.

This study has a number of limitations. First, the evidence we identified is indirect. Second, registration with the two orders of physicians may not be a highly accurate measure of actual membership of the physician workforce. Physicians who permanently or temporarily reside abroad might have kept their Lebanese registration active. Additionally, some physicians might be illegally practicing in Lebanon, especially in remote areas, without being registered. We expect these numbers to be very low, however, given the requirement to be registered to bill major payers such as the National Social Security Fund. Third, physicians may opt to delay their registration until they complete their residency training; it is thus not clear whether a delayed registration versus increasing emigration explains the relatively low percentage of registration by Lebanese medical graduates of recent years (Figure 5). Fourth, the variable "year of registration" (used in the 3^rd ^set of analysis) was not available for those registered as of 2007 with one of the two Orders. However these constituted only 12% of the total number of registered physicians. Finally, because of the nature of the administrative data, we did not plan to study the "oversaturation of the physician job market" suggested by the qualitative study and focused instead on the growth of the number of physicians. However, the leveling off of the physician density since the early 2000s (Figure 4) supports the suggestion that the market is oversaturated [3].

While the findings are consistent with earlier reports of a dramatic growth of the workforce in the early 1990s [4], they show a slowing of this growth since the early 2000s. However registration data do not clarify whether the reduction in registration rates is related to a slowing on the supply end (i.e. decreased repatriation of immigrant physicians or fewer Eastern European graduates entering the workforce) or an acceleration of emigration out of the workforce, or both. Nevertheless, there is indirect evidence that an accelerated emigration is contributing to this phenomenon. In a 2005 survey of graduating Lebanese medical students, 96% intended to travel abroad either for a specialty (78%) or a subspecialty (18%) training [7]. It is well known that residency training abroad is a critical step in the migration of physicians [8]. In addition, only 35% of survey respondents intended to return to Lebanon directly after finishing training abroad.

The Lebanese physician workforce has failed to assimilate about 40% of the country medical graduates of the last three decades. This however is not a new phenomenon. Kronfol et al. found that in 1974, 49% of the 1935–1974 medical graduates of the American University of Beirut, a Lebanese university with cultural and educational ties to the US, were in the US [9]. In 1984, 70% of graduates of the same university were in the US [10]. The author attributed this sharp increase in immigration to the US on the civil war of that time.

The rapid growth of the number of physicians can lead to a number of serious workforce problems besides emigration. These problems include unemployment, employment in other fields, overuse of services and illegal practice. Unfortunately, we were not able to locate any studies assessing these issues.

There are two major concerns with the shift in the workforce demography. First, the "oversaturation of the market" is motivating Lebanese medical graduates to emigrate [3], and is likely discouraging migrant Lebanese physicians in other parts of the world to consider resettling in Lebanon. This would likely exacerbate Lebanon's brain drain problem leading to losses in educational costs and returns on investment as described for other brain drained countries [11]. Second, it is believed that the certification process of medical graduates entering the Lebanese workforce is not rigorous enough to filter out those who received low quality training abroad, which could affect the quality of care in Lebanon.

Most registered physicians who completed specialty training did so abroad, mostly in Eastern and Western Europe. Specialty training abroad was associated with, among other factors, the region of medical school being abroad, particularly Eastern Europe. This observation raises the question of why those graduating from Eastern European medical schools are not completing residency training in Lebanon, A possible answer could be related to a problem of availability and quality of residency training in Lebanon, as students of Lebanese medical schools have suggested [3].

Northern Africa, Eastern Europe, and Western Europe as the regions of medical school and Western Europe as the region of specialty training were associated with age and male sex. Because subjects being analyzed are those currently registered in Lebanon, it is hard to judge whether those who are older (and thus graduated earlier from medical schools) or those who are male were more likely to complete specialty training abroad, or were more likely to return to Lebanon after completing specialty training in Western Europe, or both. It is however likely that females are in a weaker socio-economic position to travel abroad for medical education.

It is important to note that while non-Lebanese citizens can enter and graduate from a Lebanese medical school, they can't register or practice medicine in Lebanon. This implies that repatriating physicians would all be Lebanese citizens unlike emigrating Lebanese medical graduates. However, the implications of this phenomenon should be minimal; in a recent survey non-Lebanese citizens constituted less than 6% of students of Lebanese medical schools [7].

## Conclusion

Lebanese policy makers should not be reassured by the slowing of the growth of the workforce as this statistic may in fact reflect an increasing rate of emigration of physicians. Similarly to other countries suffering from physicians' workforce crises, there is an urgent necessity to formulate a comprehensive national health workforce plan [12]. Such a plan would need to carefully address factors contributing to the emigration of physicians such as the oversaturation of the Lebanese job market, and residency training capacity and quality. Such a plan could include defining the optimal size of the physician workforce, devising strategies to reach this size, and improving local residency training programs. Some authors have suggested a "numerus clausus" to limit the annual number of Lebanese students who enter medical schools but the effectiveness of such strategy is debatable [4].

In general, more workforce research is needed in order to generate evidence to inform policy decisions [13]. Specifically, future research should monitor the growth of the Lebanese physician workforce with special attention to specific subgroups such as Lebanese medical graduates and medical graduates of Eastern Europe. At the same time, there is a need to study the capacity and quality of residency training in Lebanon and to explore the potential association between the country/region of medical education on one hand and the quality of care provided by physicians and the socioeconomic status of these physicians on the other hand.

## Competing interests

The authors declare that they have no competing interests.

## Authors' contributions

EAA: conception and design, data collection, data analysis, data interpretation, article drafting, final approval of the article, obtaining of funding. KEA: data analysis, data interpretation, critical revision of the article, final approval of the article. BKM: data collection, data interpretation, critical revision of the article, final approval of the article, obtaining of funding. NM: data collection, data interpretation, critical revision of the article, final approval of the article. SA: data interpretation, critical revision of the article, final approval of the article, obtaining of funding.

## Pre-publication history

The pre-publication history for this paper can be accessed here:



## References

[B1] Mullan F (2005). The Metrics of the Physician Brain Drain. New England Journal of Medicine.

[B2] Akl EA, Maroun N, Major S, Chahoud B, Schünemann HJ (2007). Graduates of Lebanese medical schools in the United States: an observational study of international migration of physicians. BMC Health Services Research.

[B3] Akl EA, Maroun N, Major S, Afif C, Chahoud B, Choucair J, Sakr M, Schunemann HJ (2007). Why are you draining your brain? Factors underlying decisions of graduating Lebanese medical students to migrate. Social Science & Medicine.

[B4] Daher M, Husseini H, Kasparian R, Kasparian C (1998). Medical demography in Lebanon. Plethora, feminization, youthfulness. Lebanese Medical Journal.

[B5] Kassak K, Ghomrawi H, Osseiran A, Kobeissi H (2006). The providers of health services in Lebanon: a survey of physicians. Human Resources for Health.

[B6] United Nations Department of Economic and Social Affairs, Population Division's World Population Prospects: the 2006 Revision Population Database.

[B7] Akl EA, Maroun N, Major S, Afif C, Abdo A, Choucair J, Sakr M, Li CK, Grant BJB, Schünemann HJ (2008). Post-graduation migration intentions of students of Lebanese medical schools: a survey study. BMC public health.

[B8] Hilary J (2001). The wrong model: GATS, trade liberalisation and children's right to health.

[B9] Kronfol NM (1979). The migratory flow of the medical graduates of the American University of Beirut 1935–74 Dissertation.

[B10] Kronfol NM, Sibai AM, Rafeh N (1992). The impact of civil disturbances on the migration of physicians: the case of Lebanon. Medical Care.

[B11] Kirigia JM, Gbary AR, Muthuri LH, Nyoni J, Seddoh AT (2006). The cost of health professionals brain drain in Kenya. BMC Health Serv Res.

[B12] Hagopian A, Micek M, Vio F, Gimbel-Sherr K, Montoya P (2008). What if we decided to take care of everyone who needed treatment? Workforce planning in Mozambique using simulation of demand for HIV/AIDS care. Human Resources for Health.

[B13] El-Jardali F, Jamal D, Abdallah A, Kassak K (2007). Human resources for health planning and management in the Eastern Mediterranean region: facts, gaps and forward thinking for research and policy. Human Resources for Health.

